# Recognition and treatment of polypoidal choroidal vasculopathy and age-related macular degeneration in British Columbia

**DOI:** 10.1186/s40942-025-00744-8

**Published:** 2025-10-27

**Authors:** Yoomin Lee, Sidrat Rahman, Brendan K. Tao, Tony Fang, Handan Akil, Daniela Ferrara, Simon Lam, Eduardo V. Navajas

**Affiliations:** 1 Faculty of Medicine, McGill University, Montreal, QC Canada; 2 Department of Ophthalmology, University of Toronto, Toronto, ON Canada; 3 Department of Medicine, University of Toronto, Toronto, ON Canada; 4 Department of Ophthalmology, University of Liverpool, Liverpool, UK; 5 Department of Ophthalmology, Tufts University, Boston, MA USA; 6 Department of Ophthalmology and Visual Sciences, University of British Columbia, 2550 Willow Street, Vancouver, BC V5Z 3N9 Canada

**Keywords:** Polypoidal choroidal vasculopathy, Age-related macular degeneration, Anti-vascular endothelial growth factor, Photodynamic therapy, Indocyanine green angiography, Retrospective, Caucasian, Chinese

## Abstract

**Objective:**

To assess ethnic differences in the prevalence, diagnosis, and management of polypoidal choroidal vasculopathy (PCV) compared to typical neovascular age-related macular degeneration (nAMD) among Chinese and Caucasian patients in British Columbia.

**Methods:**

A retrospective chart review was conducted between 2008 and 2013 based on predefined inclusion criteria. Demographic, diagnostic, and clinical outcome data—including race, lesion type, diagnostic modality, and treatment response—were extracted and analyzed using RStudio.

**Results:**

Of the 416 eyes reviewed, 92.07% (383/416, N = Total sample number) were from Caucasian patients and 7.93% (33/416, N = Total sample number) from Chinese patients. No statistically significant difference in median age at diagnosis was observed between ethnic groups. ICGA was used in 3.57% (2/56, N = Total number of PCV cases) of PCV cases. Anti-VEGF monotherapy was the predominant treatment modality, while combination therapy with photodynamic therapy (PDT) was employed in 5.38% (22/353 + 56) of cases. There were no significant differences in visual acuity (VA) gains or central retinal thickness (CRT) reductions between PCV and nAMD diagnoses, nor between ethnic groups. Under anti-VEGF monotherapy, visual acuity and central retinal thickness outcomes were similar between PCV and nAMD and across the ethnic groups evaluated, within the limitations of the study design.

**Conclusion:**

PCV, though less common than typical nAMD, was identified in both Chinese and Caucasian patients, with a trend toward younger age at presentation compared to nAMD. The low utilization of ICGA may contribute to the underdiagnosis of PCV. Nevertheless, anti-VEGF monotherapy yielded comparable outcomes across diagnoses and ethnicities, underscoring the importance of equitable diagnostic and therapeutic strategies in nAMD care.

**Supplementary Information:**

The online version contains supplementary material available at 10.1186/s40942-025-00744-8.

## Background

Age-related macular degeneration (AMD) is a progressive eye disease resulting in central vision loss through changes in the vasculature of the retinal layers of the macula [1]. It is the global leading cause of vision loss in the elderly population, with the global prevalence being estimated to be 8.01% and this condition is projected to afflict 288 million patients by 2040 [2, 3]. In Canada, over 2 million patients over 50 years of age are affected by AMD, accounting for 90% of severe and irreversible vision loss [4]. This condition is associated with a diminished quality of life as AMD lowers independence and increases the risk of depression and physical injury [5].

There are two different types of AMD: dry and neovascular (also known as wet AMD) [1]. Neovascular AMD (nAMD) is characterised by abnormal proliferation of choroidal neovascular membranes (CNV) due to overexpression of vascular endothelial growth factor (VEGF) [1]. A distinct subtype of nAMD is Polypoidal choroidal vasculopathy (PCV) [6]. PCV is frequently misdiagnosed as typical nAMD and is also more prevalent among Asian than White populations [6]. To-date, the literature supports that the PCV and AMD are distinct entities, which follow different clinical courses, and require their own pathways for clinical diagnosis and management [3, 6–8]. Compared to typical nAMD, PCV is associated with greater risk of recurrent serous or hemorrhagic pigment epithelial detachments (PED), massive macular hemorrhage, and break-through vitreous hemorrhage [6]. Although nAMD typically responds to intravitreal anti-VEGF therapy, evidence indicates that the photodynamic therapy (PDT), whether alone or in combination with anti-VEGF, is an effective treatment for PCV [9, 10].

There remains a scarcity of investigation on the practical clinical differentiation between PCV and typical nAMD in the Canadian population, and this literature gap remains understudied in western Canada which harbours a significant Asian population [11]. Indocyanine green angiography (ICGA) is the gold standard tool for identification of PCV, yet from our experience in western Canada, this modality is not always utilized in patients with clinical suspicion of PCV. Herein, in this retrospective chart review, we sought to review patterns in the practical clinical differentiation of PCV from typical nAMD among Chinese and White patients in Vancouver, British Columbia, Canada. Our primary outcome was the incidence of PCV versus typical nAMD among Asian versus White patients, and the distribution of diagnostic (ICGA and/or optical coherence tomography - OCT) and treatment (anti-VEGF and/or PDT) modalities to these groups. Secondarily, we compare these demographic groups to final follow-up on change-from-baseline in visual acuity (VA), number of injections, and central retinal thickness (CRT).

## Ethics

This study abided by the tenets of the declaration of Helsinki. This study was a retrospective chart review. The research involves no direct patient contact or intervention, and all data were collected solely from existing medical records. As such, the project posed no more than minimal risk to participants. Obtaining individual consent was impracticable given the retrospective nature of the study, the extended timeframe over which the charts were generated, and the potential difficulty in re-contacting patients, some of whom may no longer be followed in clinically beyond the study evaluation period. No identifying information were retained in the research dataset, and all analyses were conducted on de-identified data. No identity identifiable images or records, which would otherwise require consent, were included in the final publication. Thus, a waiver of consent was deemed appropriate in accordance with the Tri-Council Policy Statement 2 Article 3.7, since the current work could not be feasibly conducted otherwise, and the welfare and privacy of participants were fully protected.

Regarding protection of patient privacy, data of included patients were anonymized and stored on an encrypted, password-protected drive. The identifiable patient information of all included participants were kept in a secure, encrypted, password-protected patient log file, where they were assigned a de-identified, unique, randomly generated study identifier number. This identifier number was used in a separate secure, encrypted, password-protected data extraction file. At completion of the study, the identifier log file along with identifiable patient data was destroyed, leaving only the anonymize dataset for analysis. All computers with data were password protected. All imaging data were stored securely in a password-protected file registry on the image acquisition platform. This storage is consistent with standard clinical practices at the Vancouver General Hospital Eye Care Centre. Data on patients not included in the analysis were destroyed for optimal preservation of privacy.

This retrospective chart review was accepted by the University of British Columbia Research Ethics Board (H15-01794) on September 14, 2015, and updated August 31, 2020, during which the entirety of the study involving identifiable data was completed during the approval period.

## Data availability

The data used for this study is available upon reasonable request from the corresponding author.

## Methods

The study was performed using data of the offices of 4 retinal specialists. Patient records were identified through keyword-based filtering terms such as “age-related macular degeneration” (AMD) and “polypoidal choroidal vasculopathy” (PCV). The review period extended from March 25, 2008, to June 25, 2013.

Patients were included if they met the following criteria: (i) age ≥ 65 years; (ii) a diagnosis of primary or recurrent CNV secondary to age-related macular degeneration (AMD); (iii) a documented history of treatment with intravitreal anti-VEGF agents; and (iv) no prior diagnosis of AMD before the inception of the database.

Exclusion criteria included: (i) follow-up duration of less than one year; (ii) diagnosis of secondary CNV due to causes other than neovascular AMD (nAMD); and (iii) non-Chinese or non-White race.

From each patient, data were collected on race (binarized as Chinese or White), neovascular lesion type (PCV or nAMD), diagnostic modality OCT and ICGA, and clinical outcomes through follow-up, including best-corrected visual acuity (BCVA) and central retinal thickness (CRT). In this study, VA was analyzed in Logarithm of the Minimum Angle Resolution (LogMAR) and CRT in micrometers (um). Only one eye per patient was included in the analysis. If both eyes fit the criteria, the eye with the lower BCVA was selected.

Based on multimodal imaging, classic or occult CNV on fluorescein angiography, as well as type 1 or type 2 CNV on OCT, were considered eligible for inclusion. Diagnoses of PCV by OCT were confirmed according to the tomographic criteria defined by De Salvo et al. (2014), including the presence of a sharp pigment epithelial detachment (PED) peak, PED notch, and a hypo-reflective lumen within hyper-reflective lesions adherent to the retinal pigment epithelium. These features yield a reported sensitivity of 94.6% and specificity of 92.9% for the identification of PCV lesions using OCT [12]. Diagnostic criteria for PCV on ICGA included choroidal vascular changes, such as branching vascular networks in the inner choroid and vascular dilations at the periphery of these networks [13].

Descriptive and comparative statistical analyses were conducted using RStudio (RStudio, PBC, Boston, United States of America), an integrated development environment for R. All percentages are reported as n/N (%), where N = nAMD + PCV within the specified subgroup if indicated (e.g. ethnicity), unless otherwise stated. Continuous outcomes were presented as median [IQR] with 95% confidence intervals (CI). The normality of data distributions was assessed using the Shapiro–Wilk test. Qualitative variables were summarized as ratios and calculated percentages, while quantitative variables were reported as medians with interquartile ranges (IQRs).

Comparative analyses were performed using non-parametric tests. For comparisons between two independent non-normally distributed groups, the Mann–Whitney U test (implemented as the Wilcoxon Rank-Sum test with continuity correction in RStudio) was used. For comparisons involving three or more non-parametric groups, the Kruskal–Wallis test was applied. A p-value of < 0.05 was considered statistically significant. For statistically significant results of the Kruskal-Wallis test, the Dunn’s test was done, and the p-value was adjusted depending on the number of comparisons performed.

## Results

Our study included a total of 416 individuals, with a median age at diagnosis of 82 years [IQR: 9], of which 91.4% (383/416, N = Total sample) were Caucasian and 7.9% (32/416, N = Total sample) were Chinese. The age distribution, stratified by race, is depicted in Supplemental Fig. 1. The median age at diagnosis was 82 years [IQR: 8.5] in the Caucasian group and 81 years [IQR: 11] in the Chinese group, which is depicted in Supplemental Fig. 2. Among the 416 eyes, nAMD, PCV, myopic CNV, and CSR were identified; the primary comparative analysis focused on AMD and PCV, whereas the other diagnoses were described exploratorily.

Table 1 depicts a summary of patient ethnicity and diagnosis. Of the 416 eyes analyzed, 33 were Chinese and 383 were Caucasian. In the Chinese cohort, 78.1% (25/25 + 7, N = Chinese with nAMD + PCV) were diagnosed with nAMD, and the rest with PCV. In the Caucasian cohort, the percentage of nAMD and PCV diagnoses were 87.0% (328/328 + 49, N = Caucasians with nAMD and PCV) and 13.0% (49/328 + 49), respectively. In this study, all cases of PCV were diagnosed utilizing OCT, with only 2 cases having the diagnoses confirmed by ICGA. The median age at diagnosis for both nAMD and PCV was lower in the Chinese group compared to the Caucasian group; however, no statistically significant differences were observed between the racial groups for either diagnosis. When stratified by diagnosis irrespective of race, a statistically significant difference was found between the age of diagnosis of nAMD and PCV, where AMD was diagnosed at a later age (median difference 3 years, 95% CI 1–5, *p* = 0.005), with medians 82 [IQR 8] and 79 [IQR 7] years, respectively.

**Table 1:** Baseline characteristics of the study population stratified by ethnicity and diagnosis, N = total sample number of study

	Chinese (%)	Caucasian (%)	Total (%)
nAMD	25 (6.0)	328 (78.8)	353 (84.9)
PCV	7 (1.7)	49 (11.8)	56 (13.5)
Myopic CNV	1 (0.2)	3 (0.7)	4 (1.0)
CSR	0 (0)	3 (0.7)	3 (0.7)
Total	33 (7.9)	383 (92.1)	416 (100)

A total of 22 eyes received combination therapy with photodynamic therapy (PDT) and anti–vascular endothelial growth factor (anti-VEGF) agents. Of these, 18.2% (4/22, N = Eyes that received combination therapy) were diagnosed with PCV and the remainder with nAMD. Based on diagnosis, 7.1% (4/56, N = Total number of PCV cases) of PCV patients and 5.1% (18/353, N = Total number of nAMD cases) of nAMD patients received combination therapy, while all others were treated with anti-VEGF monotherapy. Among the 409 patients with either nAMD and PCV, 1.5% were treated with Lucentis, 54.8% with Avastin and 42.1% with both (Supplemental Fig. 3). For patients with nAMD, there were no statistically significant differences between treatments in terms of change in VA or a reduction in CRT, with Kruskal–Wallis test p-values of 0.031 and 0.29, respectively. In the nAMD cohort, the Kruskal-Wallis test indicated a global difference for ∆VA (*p* = 0.031), which was not seen in CRT reduction (*p* = 0.29). However, Dunn’s multiple comparisons did not show adjusted pairwise differences for ∆VA. When the same analysis was applied to patients with both nAMD and PCV, the Kruskal–Wallis test demonstrated a statistically significant global difference in VA change (*p* = 0.024); however, subsequent pairwise comparisons using Dunn’s test did not reveal significant pairwise differences (Supplemental Fig. 4).

The change in VA, in an analysis of all the treatments together, did not differ significantly by ethnicity, with a median change of 0.301 [IQR: 1.000] in the Chinese group and 0.00 [IQR: 0.699] in the Caucasian group. This difference was not statistically significant (*p* = 0.176, HL of 0.169, 95% CI – 0.028 to 0.398). A similar finding was observed when comparing BCVA change between nAMD and PCV diagnoses (*p* = 0.644, HL of 0.000, 95% CI − 0.097 to 0.176). Furthermore, no statistically significant differences were observed when treatment modality (combination therapy with PDT vs. anti-VEGF alone) was analyzed by diagnosis (*p* = 0.116, HL of -0.155, 95% CI -0.463 to 0.000).

Changes in reduction CRT were compared between the two racial groups, without differentiating the treatment. The median change in CRT was − 28 μm [IQR: 97.0] in the Chinese group and − 80.5 μm [IQR: 127.6] in the Caucasian group, with a statistically significant difference (*p* = 0.049, HL of 42.000, 95% CI 0.000 to 87.000). When classified by diagnosis, the median change in CRT was − 80 μm [IQR: 131.5] for nAMD and − 65.5 μm [IQR: 109.25] for PCV (*p* = 0.966, HL of -1.000, 95% CI -32.000 to 32.000).

## Discussion

In this study, we investigated ethnic differences in the prevalence, diagnosis, and outcomes of PCV versus nAMD in a cohort in Vancouver, Canada. We found that 21.9% (7/25 + 7) of Chinese eyes and 13.0% (49/328 + 49) of Caucasian eyes with exudative AMD were diagnosed as PCV. Though there was a higher incidence in the Chinese patients, it was not statistically significant (*p* = 0.112). This trend aligns with the well-documented higher prevalence of PCV among Asian populations. In clinical cohorts, PCV comprises roughly 50–60% of nAMD cases in Asian patients, but only about 8–20% in individuals of European descent [2, 3]. Our study may have been underpowered to detect a significant ethnic disparity given the relatively small Chinese cohort. Nevertheless, the observed proportions highlight that PCV is not uncommon, even in Caucasian patients. We also noted that PCV patients presented at a younger median age than those with typical nAMD (79 vs. 82 years, *p* = 0.005). This finding is consistent with reports that PCV tends to affect a slightly younger demographic compared to classic nAMD [4]. Importantly, patient age did not differ by ethnicity in our cohort, suggesting that Chinese and Caucasian patients had a similar age of onset when diagnosed with late nAMD.

We also observed the underuse of ICGA in routine diagnostics in our study. Despite ICGA being the gold standard for identifying polypoidal lesions, only 3.57% (2/56, N = Total number of PCV) of eyes with PCV in our series underwent ICGA confirmation. Instead, most PCV diagnoses were presumptively made using OCT findings and clinical exam. Our findings also are consistent with the patterns reported by Chen et al. in Toronto, where they found that ICGA was infrequently used in Canadian AMD clinics. Consequently, PCV was likely under-recognized, especially among Caucasian patients [6]. Chen et al. [6] also reported that only 0.7% of newly diagnosed Caucasian nAMD eyes were identified as PCV versus 6.5% in Chinese eyes and they concluded that PCV was being overlooked in many Caucasian patients, largely due to limited use of ICGA.

In our cohort, however, a considerably higher portion of Caucasian nAMD cases (13.0%) were diagnosed as PCV, which may reflect an increased clinical suspicion and reliance on OCT-based hallmarks of PCV. In fact, prior studies have shown that experienced clinicians can detect PCV via characteristic OCT features with high accuracy. De Salvo et al. [7] described OCT signs such as a “sharp-peaked” pigment epithelial detachment (PED), notched or multilobular PEDs, and hyperreflective sub-RPE ring-like lesions that distinguish PCV from typical occult CNV. In a 2014 study, they demonstrated that these OCT features could identify PCV with 94.6% sensitivity and 92.9% specificity compared to ICGA [7]. Additional consensus guidelines from the Asia-Pacific Ocular Imaging Society PCV Workgroup further validated these non-invasive diagnostic features, highlighting their utility when ICGA is unavailable [8]. Such data suggest that careful OCT analysis can often obviate the need for ICGA in diagnosing PCV. In our center, ophthalmologists likely leveraged these non-invasive clues, allowing them to recognize PCV in many patients despite the rareness of ICGA, albeit at the risk of some misclassification. This diagnostic approach reflects real-world constraints and reinforces the need for improved access to definitive imaging or validated OCT criteria to ensure PCV is identified across all ethnic groups [9–11].

Regarding the treatment patterns, nearly all patients in our study received anti-VEGF monotherapy, while only 5.4% (22/410, N = AMD + PCV of both ethnicities) underwent combination therapy with photodynamic therapy (PDT) plus anti-VEGF. In our study, the majority of patients with nAMD were treated with Avastin only, followed by those receiving alternating monotherapy with Lucentis and Avastin. In contrast, all patients with PCV had alternating monotherapy with Lucentis and Avastin. Eylea was not utilized because it was covered during the time the study was conduct.

In the context of this study sample, no differences in visual acuity and CRT reduction were found for this cohort of patients of nAMD and a cohort with both nAMD and PCV. Our findings were consistent with those reported by Cho et al. [14] for PCV and Berg et al. [15] for nAMD. In patients with nAMD, Berg et al.^15^ reported that after two years of treatment with bevacizumab (Avastin) and ranibizumab (Lucentis), both therapies yielded comparable improvements. Similar findings were observed in PCV patients, where both treatments produced equivalent gains in visual acuity and reductions in macular edema [14]. However, ranibizumab may offer a slight advantage in its ability to reduce exudation [14].

This indicates that even among eyes suspected to have PCV, most were managed by injections alone. The low utilization of PDT in our setting may stem from limited availability of PDT in North America and the prevailing confidence in anti-VEGF agents as first-line therapy for nAMD, including PCV [12, 13]. It is remarkable that our monotherapy-heavy approach did not lead to inferior outcomes in the short term. We observed no significant differences in visual acuity or central retinal thickness improvements between eyes with PCV and those with typical nAMD. This suggests that, at least over the follow-up period analyzed, anti-VEGF monotherapy was effective in controlling exudation and maintaining vision in PCV patients to a degree comparable with conventional nAMD treatment. Our findings are in line with major clinical trials that have evaluated therapies for PCV. For example, the EVEREST trial demonstrated that while adjunctive PDT produces superior polyp regression (with polyp closure rates ∼ 77–78% when PDT is used, versus ∼ 29% with ranibizumab alone), the 6-month visual gains were comparable among eyes treated with anti-VEGF monotherapy versus combination therapy [16]. More recently, EVEREST II reported that combination therapy (ranibizumab + PDT) achieved a slightly greater vision improvement at 12 months than ranibizumab alone (+ 8.3 vs. + 5.1 ETDRS letters, *p* = 0.013) along with significantly higher polyp closure (69.3% vs. 34.7%) [17]. In contrast, the PLANET study, which examined aflibercept in Asian PCV patients, found that the vast majority (> 85%) attained substantial visual gains with aflibercept monotherapy, and adding PDT (as rescue therapy) provided no significant additional benefit in those first 12 months [18]. Additional studies have also confirmed the efficacy of anti-VEGF monotherapy while acknowledging a potential benefit of combination therapy for select PCV phenotypes [19–21].

Importantly, our analysis did not reveal any significant disparities in treatment outcomes between Chinese and Caucasian patients. Both ethnic groups achieved comparable mean visual acuity gains and central retinal thickness reductions at follow-up. This is an encouraging indication that, given similar management, patients have equitable outcomes regardless of ethnicity. It suggests that the biological response to anti-VEGF therapy and the prognosis of nAMD/PCV were similar in our Chinese and Caucasian subgroups. This also implies that it is the tailored management of PCV that can ultimately influence the outcomes.

## Possible limitations

There are several limitations to our study. First, its retrospective nature and reliance on chart documentation may introduce selection bias, as not all eligible patients may not have been represented. Misclassification bias is also possible as some PCV cases might have been missed or misclassified due to the lack of confirmatory ICGA for most patients. We attempted to mitigate this by using established OCT criteria and clinical correlation, but diagnostic uncertainty remains a possibility. Second, the sample size of the Chinese subgroup was relatively small (33 eyes, 7 diagnosed PCV), which limits the statistical power to detect subtle differences between ethnic groups. A larger cohort might confirm an ethnicity-related difference in PCV prevalence that our study was underpowered to show. Additionally, our follow-up duration was limited to the available data (with a minimum of one year in most cases). Thus, longer-term outcomes such as recurrence rates, scar formation, or vision at 5 years were beyond the scope of this review. Lastly, the study was conducted using data provided by 4 retina specialists that practice both in a tertiary centre and community areas in a large city that has a considerable Caucasian and Chinese population. As a result, this study supports generalizability to similar urban populations with analogous demographics, but not beyond those groups. Additionally, the patient demographics and physician practice patterns here (for instance, comfort with OCT-based diagnosis or access to PDT) may differ from other regions. Therefore, caution is warranted in generalizing these findings to broader populations. Additionally, the referral pattern to tertiary and retina clinics may overrepresent severe cases, introducing potential referral bias. Despite these limitations, our study offers insight into real-world management of PCV vs. nAMD in a multi-ethnic North American context, a topic on which data have been relatively sparse.

## Conclusion

In conclusion, PCV was observed in a substantial proportion of both Chinese and Caucasian patients, and outcomes with anti-VEGF therapy were comparable across groups. Given the low use of ICGA and PDT, there is a need to enhance diagnostic precision and tailor treatment strategies based on PCV features, regardless of ethnicity. Improved access to imaging, greater awareness among clinicians, and prospective multicenter studies are necessary to ensure equitable, optimized care for all patients with PCV.

## Supplementary Information

Below is the link to the electronic supplementary material.

Supplementary Material 1

## Data Availability

The data used for this study is available upon reasonable request from the corresponding author.

## Abbreviations

AMD: Age-Related Macular Degeneration
BCVA: Best-Corrected Visual Acuity
CNV: Choroidal Neovascular Membranes
CRT: Central Retinal Thickness
CSR: Central Serous Retinopathy
ETDRS: Early Treatment Diabetic Retinopathy Study
ICGA: Indocyanine Green Angiography
IQR: Interquartile Range
logMAR: Logarithm of the Minimum Angle of Resolution
nAMD: Neovascular AMD
OCT: Optical Coherence Tomography
PCV: Polypoidal Choroidal Vasculopathy
PED: Pigment Epithelial Detachments
PDT: Photodynamic therapy
SANGRA: Surname Analysis for Recognizing Chinese Ethnicity
VA: Visual Acuity
VEGF: Vascular Endothelial Growth Factor
