# Supplementary material for: Recognition and treatment of polypoidal choroidal vasculopathy and age-related macular degeneration in British Columbia
Source: Int J Retina Vitreous. 2025 Oct 27;11:118. doi: 10.1186/s40942-025-00744-8 (PMC12560305; doi:10.1186/s40942-025-00744-8)
Supplement: Supplementary file 1 — Supplementary Material 1 [file 40942_2025_744_MOESM1_ESM.docx]

**Supplementary Material**

**~~
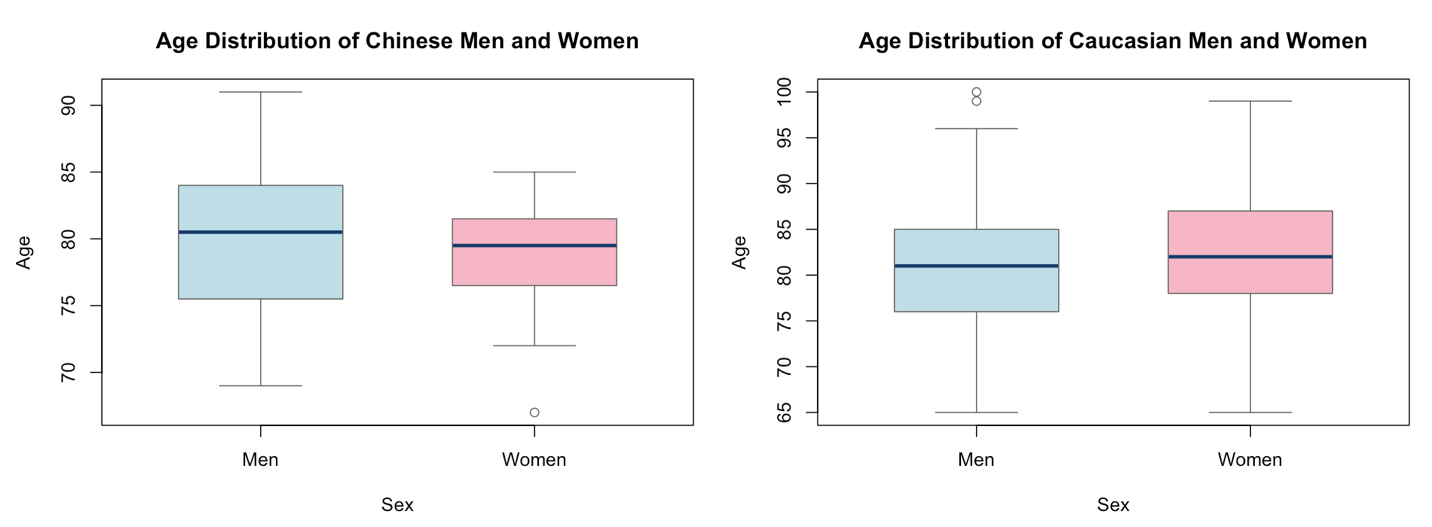
~~**

**Supplementary Figure 1.** Age Comparison of Sexes of the Sample Population Based on Race.

**
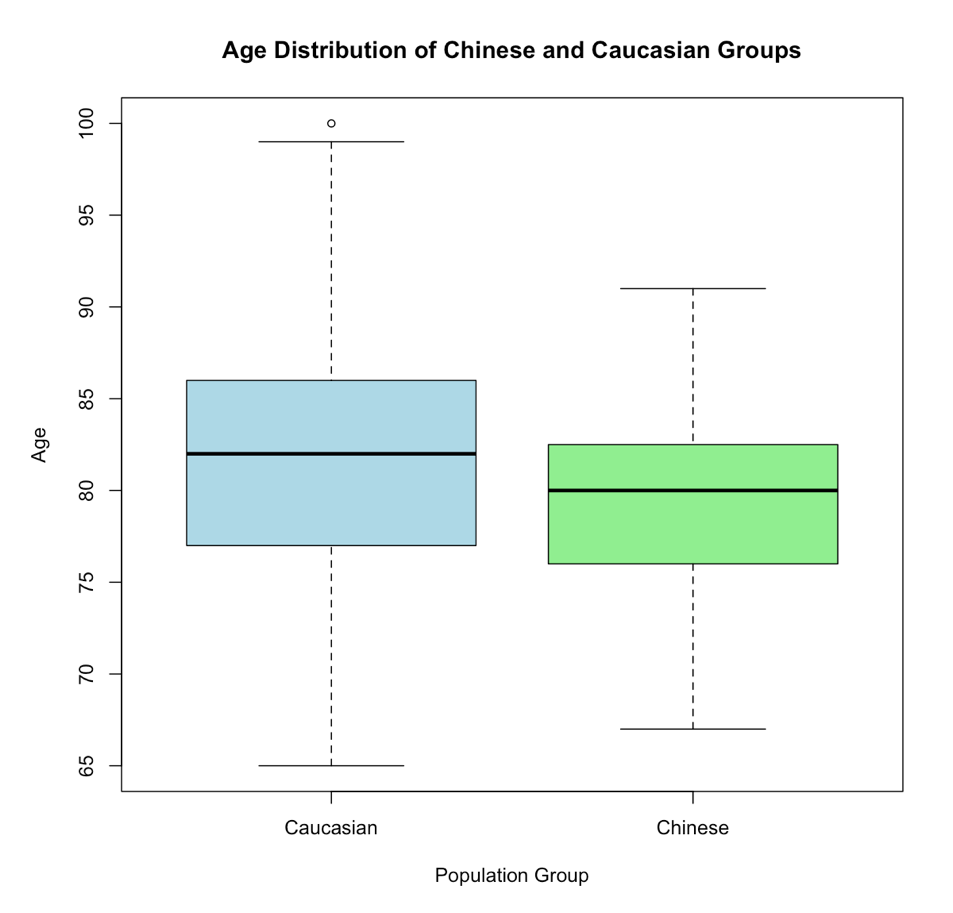
**

**Supplementary Figure 2.** Age Comparison of the Difference Races of the Sample Population.

**
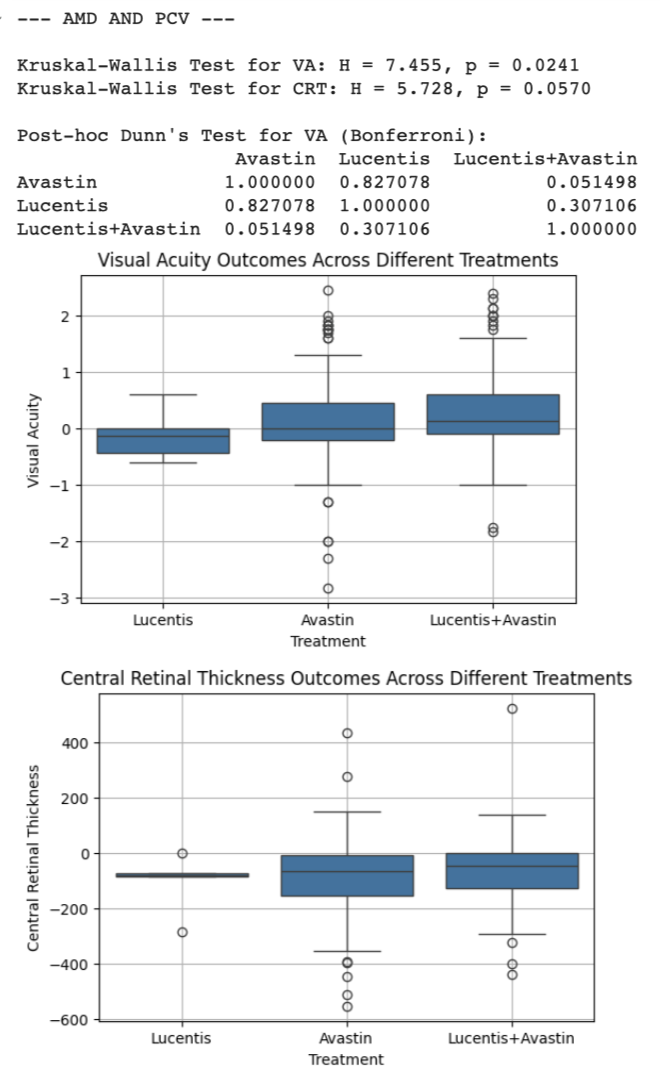
**

**Supplementary Figure 3.** Comparison of Effects of Treatment on Patients with AMD and PCV.

**
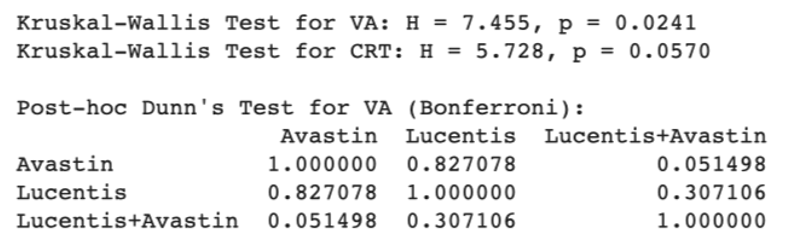
**

**Supplementary Figure 4.** Results of the Statistical Analysis for Comparing Treatments for Patients with AMD and PCV.
